# Peroxisome Proliferator-Activated Receptor *γ* and PGC-1*α* in Cancer: Dual Actions as Tumor Promoter and Suppressor

**DOI:** 10.1155/2018/6727421

**Published:** 2018-01-21

**Authors:** Seong-Hoon Yun, Sang-Heum Han, Joo-In Park

**Affiliations:** Department of Biochemistry, Dong-A University College of Medicine, Busan, Republic of Korea

## Abstract

Peroxisome proliferator-activated receptor *γ* (PPAR*γ*) is part of a nuclear receptor superfamily that regulates gene expression involved in cell differentiation, proliferation, immune/inflammation response, and lipid metabolism. PPAR*γ* coactivator-1*α* (PGC-1*α*), initially identified as a PPAR*γ*-interacting protein, is an important regulator of diverse metabolic pathways, such as oxidative metabolism and energy homeostasis. The role of PGC-1*α* in diabetes, neurodegeneration, and cardiovascular disease is particularly well known. PGC-1*α* is also now known to play important roles in cancer, independent of the role of PPAR*γ* in cancer. Though many researchers have studied the expression and clinical implications of PPAR*γ* and PGC-1*α* in cancer, there are still many controversies about the role of PPAR*γ* and PGC-1*α* in cancer. This review examines and summarizes some recent data on the role and action mechanisms of PPAR*γ* and PGC-1*α* in cancer, respectively, particularly the recent progress in understanding the role of PPAR*γ* in several cancers since our review was published in 2012.

## 1. Introduction

**Figure 1:**
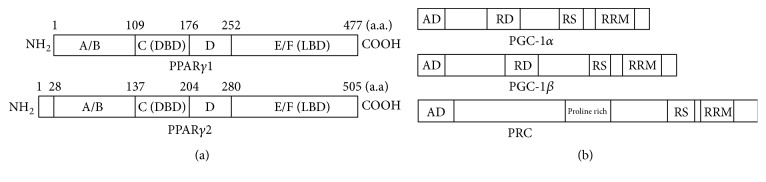
Structure of PPAR*γ* (a) and the PGC-1 family (b). (a) A/B, transcriptional activation domain; C, DNA binding domain (DBD); D, hinge region; E/F, ligand binding domain (LBD). (b) AD, transcriptional activation domain; RD, transcriptional repression domain; RS, arginine/serine rich domain; RRM, RNA binding domain.

Peroxisome proliferator-activated receptor *γ* (PPAR*γ*) belongs to a nuclear hormone receptor superfamily that regulates the expression of genes involved in cell differentiation, proliferation, the immune/inflammation response, and lipid metabolism [1]. Ligand binding and activation of PPAR*γ* result in heterodimer formation with the retinoid X receptor (RXR) and binding to a PPAR response element (PPRE) to regulate the transcription of numerous target genes [2, 3]. PPAR*γ* consists of a ligand-independent transcriptional activation domain, DNA binding domain (DBD), hinge region for cofactor docking, and ligand binding domain (LBD) (Figure 1(a)). Two PPAR*γ* isoforms are known, PPAR*γ*1 and PPAR*γ*2 [4, 5]. PPAR*γ*2, which is generated by alternative splicing, contains an additional 28 amino acids in mice and 30 amino acids in humans, at the N-terminus compared to PPAR*γ*1. PPAR*γ*2 is expressed selectively in adipose tissue and plays an important role in adipocyte differentiation, lipid storage in white adipose tissue, and energy dissipation in brown adipose tissue [4, 6]. PPAR*γ*1 is expressed in the colon, immune system, and hematopoietic cells and plays an important role in the control of inflammation, macrophage maturation, and embryo implantation. PPAR*γ*1 is a molecular target of antidiabetic thiazolidinediones [7, 8]. Our previous review summarized the role and action mechanisms of PPAR*γ* in colorectal cancer [8], but the role of PPAR*γ* in cancer is still debated. Thus, this review updates the progress in understanding the role and molecular mechanisms of PPAR*γ* in cancer.

The PPAR*γ* coactivator-1 (PGC-1) family is composed of PGC-1*α*, PGC-1*β*, and PGC-1-related coactivator (PRC). PGC-1*α* was initially identified as a transcriptional coactivator involved in mitochondrial function and thermogenesis in brown fat [9]. PGC-1*β* and PRC were discovered in sequence homology searches [10–13]. The PGC-1 family members have similar activity to increase mitochondrial function when overexpressed and have a related modular structure (Figure 1(b)). The most common functional domains are shared between PGC-1*α* and PGC-1*β*. The N-terminal activation domain interacts with several transcriptional coactivators, including p300 and steroid receptor coactivator-1 (SRC-1). A domain involved in inhibition of PGC-1 activity is located adjacent to the N-terminal region. Through several LXXLL motifs, the N-terminal half of PGC-1 interacts with many transcription factors, whereas the C-terminal end of PGC-1 interacts with the TRAP/DRIP/Mediator complex. PGC-1*α* has a Ser/Arg-rich domain and RNA binding motif that plays an important role in mRNA splicing [14, 15]. Because PGC-1*α* was described initially as a PPAR*γ* interacting protein, some investigators recently studied the expression and clinical significance of PGC-1*α* in cancer [16, 17]. However, the expression and the roles of PGC-1*α* in cancer were not significantly related to the expression of PPAR*γ*. In addition, controversies still exist whether PGC-1*α* acts as a tumor promoter or a tumor suppressor in cancer. This review focuses on the expression and actions of PGC-1*α* in order to understand the clinical significance of PGC-1*α* expression in cancer.

## 2. The Role and Action Mechanisms of PPAR***γ*** in Cancer

PPAR*γ* is expressed in various malignant tissues, including bladder, colon, prostate, and breast cancer [18–22]. Natural ligands that activate PPAR*γ* include long-chain polyunsaturated fatty acids, eicosanoids, components of oxidized low density lipoproteins (oxLDLs), and 15-deoxy-Δ^12,14^-prostaglandin J_2_ (15d-PGJ_2_) [23]. Synthetic ligands include the antidiabetic thiazolidinedione (TZD) class of drugs [23]. An increasing number of studies have focused on the effect of PPAR*γ* in cancer using natural and synthetic ligands for PPAR*γ* and overexpression experiments. However, the role of PPAR*γ* in cancer is still debated. Thus, this review updates the role and action mechanisms of PPAR*γ* in cancer since our review published in 2012.

### 2.1. PPAR*γ* as a Tumor Suppressor in Cancer

**Figure 2:**
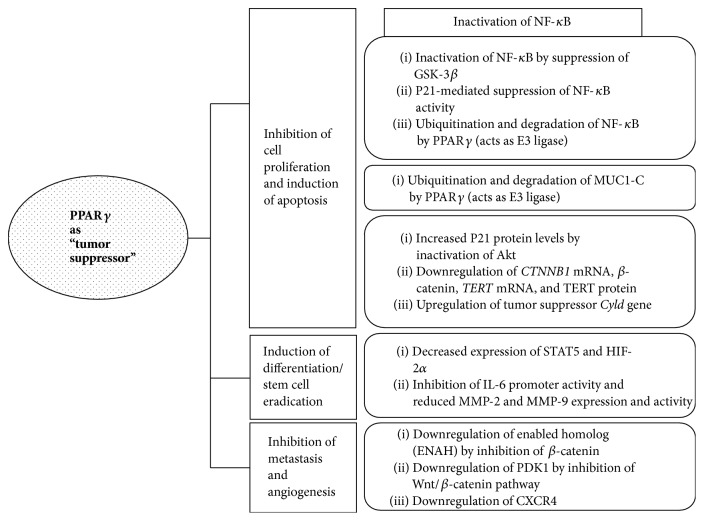
Action mechanisms of PPAR*γ* as a tumor suppressor. NF-*κ*B, nuclear factor-*κ*B; GSK-3*β*, glycogen synthase kinase 3-*β*; MUC1-C, mucin 1-C; TERT, telomerase reverse transcriptase; STAT5, signal transducer and activator of transcription factor 5; HIF-2*α*, hypoxia inducible factor-2*α*; IL-6, interleukin-6; PDK1, pyruvate dehydrogenase kinase 1.

Our previous review summarized that PPAR*γ* inhibits cell proliferation and induces apoptosis through the upregulation of Phosphatase and Tensin Homolog (PTEN), downregulation of survivin, downregulation of X-linked inhibitor of apoptosis (XIAP), suppression of NF-*κ*B and glycogen synthase kinase (GSK)-3*β*, upregulation of cyclin-dependent kinase (CDK) inhibitors, downregulation of CDK and cyclin D1, downregulation of COX-2, upregulation of Krüppel-Like Factor 4 (KLF4), upregulation of Bax, downregulation of Bcl-2, and inhibition of telomerase activity and hTERT expression through modulation of the Myc/Mad/Max network [8]. This review briefly describes and summarizes new molecular mechanisms of PPAR*γ*-related tumor suppression since 2012 (Table 1, Figure 2).

Understanding the role of PPAR*γ* in cancer was improved by developing new synthetic and natural ligands of PPAR*γ* and performing overexpression and knockdown experiments. PPAR*γ* agonist troglitazone inhibits colon cancer cell growth through the inactivation of NF-*κ*B by suppressing GSK-3*β* activity [24]. Emerging data suggest that PPAR*γ* acts as a tumor suppressor by inactivating NF-*κ*B through different mechanisms. For example, Lee et al. demonstrated that 4-O-methylhonokiol (MH), a PPAR*γ* agonist, has antitumor activity in prostate cancer through increased PPAR*γ* activity and p21-mediated suppression of NF-*κ*B activity as observed by the loss of MH-induced growth inhibition and NF-*κ*B inhibition in a p21 siRNA knockdown experiment [25]. In addition, overexpression of PPAR*γ* was shown to inhibit cell proliferation and tumor growth via degradation of NF-*κ*B by acting as an E3 ligase [26]. Hou et al. demonstrated that PPAR*γ* inhibits mucin 1- (MUC1-) C-mediated cell proliferation via MUC1-C ubiquitination and degradation [27]. MUC1-C is known as an oncoprotein and interacts with I*κ*B kinase, NF-*κ*B/p65, and signal transducer and activator of transcription factor 3 (Stat3), p53, or BAX in order to activate the downstream pathway associated with tumor growth [47–52]. Efatutazone, a third-generation PPAR*γ* agonist, has been reported to inhibit esophageal squamous cell carcinoma (ESCC) cell proliferation* in vitro* and* in vivo* through increased p21Cip protein levels via inactivation of Akt [28].

Several recent studies have shown that PPAR*γ* agonists inhibit the survival of cancer stem cells (CSCs) [29, 30, 53–55]. PPAR*γ* and RXR agonists were demonstrated to inhibit interleukin-6 (IL-6) promoter activity and reduce MMP-2 and MMP-9 expression and activity in tumor-associated fibroblasts [29]. Prost et al. demonstrated that pioglitazone, a PPAR*γ* agonist, eradicates CSCs via the decreased expression of STAT5 and HIF-2*α* in chronic myeloid leukemia [30].

The Wnt/*β*-catenin signaling pathway plays an important role in the occurrence and development of cancer [56, 57]. Guo et al. reported that PPAR*γ* overexpression inhibits the proliferation and migration of gastric cancer cells through downregulation of telomerase reverse transcriptase (TERT) and enabled homolog (ENAH) via inhibition of *β*-catenin [31]. Mammalian enabled (Mena), encoded by* ENAH*, is an actin-regulatory protein involved in controlling cell motility and cell-cell adhesion, which are important for the development of metastatic potential [58].* TERT* and* ENAH *are new targets of the Wnt/*β*-catenin signaling pathway [59, 60]. Recently, activation of canonical Wnt signaling was reported to directly act on aerobic glycolysis and increase vessel formation in colon cancer through the Wnt target gene pyruvate dehydrogenase kinase 1* (PDK1)* [61]. Via PDK1 activation, pyruvate is converted into acetyl-CoA, which enters the TCA cycle and is converted into citrate, which stimulates protein synthesis. Accumulation of metabolic intermediates (such as aspartate, glycine, serine, and ribose) in cells promotes de novo nucleotide synthesis, contributing to growth and proliferation [62]. In addition, blocking the Wnt pathway decreases PDK1 expression via transcriptional regulation and inhibits* in vivo* tumor growth [61].

Pseftogas et al. reported that PPAR*γ* activation has a tumor suppressive effect by upregulating the expression of tumor suppressor* Cyld*, as the* Cyld* promoter has PPAR*γ* binding sites [32].* Cyld* was identified as a tumor suppressor gene that is causally associated with the development of inherited cylindromas [63]. The gene encodes a protein (CYLD) possessing a carboxyl-terminal ubiquitin-specific protease domain that selectively hydrolyzes K63- and M1-linked polyubiquitin chains [64]. A number of studies have suggested a role for CYLD in the growth suppression of different types of cancer cells, such as colon, hepatocellular, lung, melanoma, and breast cancer (reviewed in [65]). CYLD can inhibit several growth and antiapoptotic signaling pathways, including the NF-*κ*B, JNK, p38, Wnt, Akt, and Notch pathways [65].

Rovito et al. demonstrated that PPAR*γ* activation downregulates CXCR4 gene expression through recruitment of the silencing mediator of retinoid and thyroid hormone receptor (SMRT) corepressor to PPRE within the CXCR4 promoter and then inhibits breast cancer cell migration and invasion [33]. CXCR4, a seven-transmembrane G-protein-coupled receptor for stromal-cell derived factor-1*α* (SDF-1*α*), has been shown to be expressed in human breast cancer cells, and activation of the SDF-1*α*/CXCR4 axis is important in breast cancer migration and metastasis [66, 67].

### 2.2. PPAR*γ* as a Tumor Promoter in Cancer

**Figure 3:**
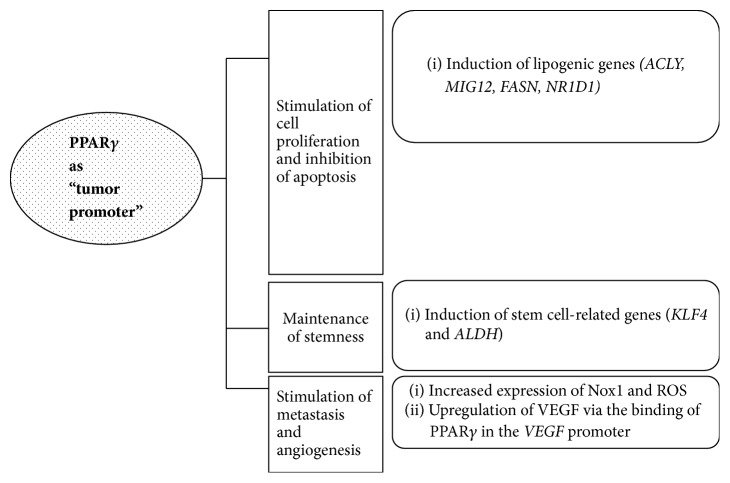
Action mechanisms of PPAR*γ* as a tumor promoter. ACLY, ATP citrate lyase; MIG12, midline-1-interacting G12-like protein; FASN, fatty acid synthase; NR1D1, Rev-ErbA*α*; KLF4, Krüppel-Like Factor 4; ALDH, aldehyde dehydrogenase; Nox1, NADPH oxidase 1; ROS, reactive oxygen species; VEGF, vascular endothelial growth factor.

Our previous review described that PPAR*γ* has tumor-promoting activity through the upregulation of *β*-catenin and c-Myc expression, upregulation of COX-2, upregulation of the expression of vascular endothelial growth factor (VEGF) and VEGF receptors, and upregulation of MMP-1 [8]. This review briefly introduces the action mechanisms of PPAR*γ* as a tumor promoter (Figure 3).

Recently, increasing evidence has indicated that PPAR*γ* acts as a tumor promoter [68–74]. Downregulation of PPAR*γ* by siRNA knockdown or treatment with PPAR*γ* antagonist GW9662 has been shown to inhibit the growth of cancer cells, suggesting a tumor-promoting effect for PPAR*γ* in these cells [68–70]. PPAR*γ* was shown to protect ErbB2-positive breast cancer cells from palmitate-induced toxicity [75]. In addition, PPAR*γ* was demonstrated to play a crucial role in the maintenance of stemness in ErbB2-positive breast cancer cells; PPAR*γ* antagonist GW9662 induces apoptosis and inhibits tumorsphere formation and tumor formation through the inhibition of lipogenic genes* (ACLY, MIG12, FASN, *and* NR1D1)* and stem cell-related genes (*KLF4 *and* ALDH*) [71]. CSCs have been identified as subpopulations of cells within tumors that promote tumor growth and recurrence [76–78].

Kesanakurti et al. demonstrated that PPAR*γ* is involved in radiation-induced epithelial-to-mesenchymal transition (EMT) in glioma by interacting with p21-activated kinase 4 (PAK4), resulting in increased Nox1 expression and reactive oxygen species (ROS) [72]. EMT is a developmental transdifferentiation program facilitating the formation of highly motile cells with stem cell characteristics [79, 80]. EMT is also involved in increased metastatic potential and treatment resistance in cancer [81, 82]. The PAKs are a family of serine/threonine kinases involved in embryonic development, cytoskeletal remodeling, cell motility, and cell proliferation [83, 84], and aberrant expression of PAK4 has been shown to promote cancer cell proliferation and invasion [85–87].

A recent study using PPAR*γ* siRNA showed that PPAR*γ* suppression inhibits cell proliferation, colony formation, and tumorigenicity* in vivo* [73]. In addition, PPAR*γ* upregulated VEGF expression through the binding of PPAR*γ* in the promoter region of* VEGF* in prostate cancer cells [73]. Patitucci et al. demonstrated that PPAR*γ* activation is involved in steatosis-associated liver cancer and provided evidence supporting the pharmacological modulation of hepatic PPAR*γ* activity as a therapeutically relevant strategy in hepatic malignancy associated with activated Akt2 and PPAR*γ* signaling [74].

## 3. The Role and Action Mechanisms of PGC-1***α*** in Cancer

Many studies have examined the role of PGC-1*α* in cancer by observing its expression in several cancers and performing PGC-1*α* overexpression and siRNA knockdown experiments. PGC-1*α* expression has been shown in some studies to be decreased in some types of cancer, including colon [88], breast [89], and ovarian cancer [41], whereas other studies have shown that PGC-1*α* expression is increased in cancer [17, 90]. Even though many studies have been published, the role of PGC-1*α* in cancer is still controversial. Therefore, this review describes the role and action mechanisms of PGC-1*α* in cancer (Table 2).

### 3.1. Tumor-Promoting Functions of PGC-1*α*

As described above, PGC-1*α* is a regulator of PPAR*γ* activity. Thus, the abnormalities in PGC-1*α* expression may affect PPAR*γ* function. However, there was little report supporting that PGC-1*α* expression directs PPAR*γ* activity in cancer. Thus, this review focuses on the role of PGC-1*α*, independent of the role of PPAR*γ* in cancer. Literature works supporting the tumor-promoting functions of PGC-1*α* have increased [17, 34–40, 42, 91–93]. Shiota et al. showed that PGC-1*α* promotes cell growth through the activation of androgen receptor in prostate cancer cells by observing cell growth inhibition with PGC-1*α* knockdown experiments [17]. In addition, PGC-1*α* was increased in tumor samples from arsenic-induced skin cancer patients and may be associated with increased cell proliferation and enhanced mitochondrial biogenesis [34]. Bhalla et al. showed that PGC-1*α* promotes carcinogenesis and tumor growth through the induction of lipogenic enzymes (acetyl-CoA carboxylase and fatty acid synthase) using genetically modified PGC-1*α* mice [35]. That study demonstrated that PGC-1*α* knockout mice had decreased chemically induced liver and colon carcinogenesis, suggesting that PGC-1*α* may stimulate carcinogenesis [35]. Similarly, Shin et al. first demonstrated that overexpression of PGC-1*α* enhances cell proliferation and tumorigenesis via the upregulation of Sp1 and acyl-CoA binding protein [36]. It was also reported that PGC-1*α* overexpression leads to increased antioxidant enzymes (catalase, superoxide dismutase) and decreased ROS-induced apoptosis [36]. Similarly, PGC-1*α* knockdown significantly decreased cell number and induced apoptosis in PGC-1*α* positive melanoma cell lines, suggesting that PGC-1*α* is crucial in the survival of PGC-1*α* positive melanoma cells [37]. In addition, superoxide dismutase 2 protein levels were decreased in PGC-1*α* depleted melanoma cells. Moreover, ectopic expression of PGC-1*α* in melanoma cells increased the expression of ROS detoxifying genes. These data support the hypothesis that PGC-1*α* plays an important role in activating the ROS detoxification gene program to maintain melanoma cell survival [37]. Vazquez et al. also demonstrated that there was a significant reduction in tumor size in PGC-1*α* depleted cells, implying PGC-1*α* may be important in tumor progression [37]. De novo lipogenesis is a distinctive anabolic feature of malignant cells [94]. Carbons from glucose and glutamine supply cytoplasmic citrate for fatty acid synthesis with the help of lipogenic enzymes [94]. Glutamine can serve as an anaplerotic mitochondrial fuel and seems to be important for tumor survival [95]. In ErbB2-positive breast cancer cells, the PGC-1*α*/ERR*α* complex directly regulates the expression of glutamine metabolism enzymes, leading to the provision of glutamine carbons to de novo fatty acid synthesis [38]. PGC-1*α* overexpression, or ERR*α* activation, confers growth advantages of breast cancer cells even under limited nutrients, supporting the correlative clinical data that high expression of PGC-1*α* is associated with poor prognosis, possibly related to the activation of its downstream glutamine pathway target genes [38]. It was reported that PGC-1*α* expression is affected by various transcriptional pathways. One example is that melanocyte-lineage transcription master regulator and oncogene MITF activated PGC-1*α* expression in melanoma [37, 91]. The decrease in mitochondrial membrane potential and increased ROS production with a decrease in glutathione, cystathionine, and 5-adenosylhomocysteine were observed in PGC-1*α*-depleted melanoma cell lines, suggesting that intrinsic apoptotic pathway is activated in PGC-1*α*-depleted melanoma cells [37]. Another example is that the androgen receptor-AMP-activated protein kinase (AMPK) signaling axis increased expression of PGC-1*α* to drive growth advantages in prostate cancers [39]. It was also shown that PGC-1*α* expression was significantly higher in lung adenocarcinomas with wild type p53 than in tumors with mutant p53 [40]. Cell proliferation was inhibited by PGC-1*α* siRNA knockdown experiments in H1944 lung adenocarcinoma cells [40]. In metabolic stress conditions, PGC-1*α* was shown, in complex with p53, to coactivate the transcription of cell cycle inhibitors, while it was also shown to promote the expression of genes related to mitochondrial biogenesis. These two functions are cooperative processes that promote cell survival. Moreover, oxidative stress in PGC-1*α* knockdown cells resulted in p53-induced apoptosis [96]. In turn, it was also shown that increased expression of PGC-1*α* might prevent p53-induced cell death by maintaining an adequate balance between oxidative phosphorylation and glycolysis [97].

Some studies have examined the effect of PGC-1*α* on angiogenesis. PGC-1*α* has been reported to activate the production of VEGF through the estrogen-related receptor *α*- (ERR*α*-) dependent pathway [98]. PGC-1*α* was shown to regulate HIF-1*α* activity. Increased PGC-1*α* expression enhances oxygen consumption, resulting in decreased local oxygen tension and increased HIF-1*α* stability [99]. In addition, HIF-2*α* is a transcriptional target of PGC-1*α*, even though the involved transcriptional mechanism is not clear [100]. ERR*α* is overexpressed in many cancers and its inhibition reduces cell proliferation. Recent studies suggest an important role for the interaction between PGC-1*α* and ERR*α* in cancer (reviewed in [15]). Kinase suppressor of ras 1 (KSR1), a molecular scaffold of the Raf/MEK/extracellular signal-regulated kinase (ERK) cascade, has been demonstrated to promote oncogenic Ras-dependent anchorage-independent growth through the activation of PGC-1*α* and ERR*α* [92]. Interestingly, recent study shows that PGC-1*α* plays an important role in the metastatic switch. LeBleu et al. demonstrated that circulating mammary epithelial cancer cells exhibit increased PGC-1*α* expression, enhanced mitochondrial biogenesis, and oxidative phosphorylation, which may contribute to distant metastasis and poor patient outcome [93]. In addition, PGC-1*α* knockdown decreased ATP production, reduced actin cytoskeleton remodeling, lowered anchorage-independent survival, and decreased intra-/extravasation, which are all checkpoints that prevent metastasis in MDA-MB-231 breast cancer and B16F10 melanoma cells [93]. LeBleu et al. also showed that PGC-1*α* expression in invasive cancer cells was significantly associated with the formation of distant metastases in a clinical analysis of human invasive breast cancers [93].

### 3.2. Anticancer Functions of PGC-1*α*

As opposed to the tumor-promoting role of PGC-1*α* described above, several studies have shown that PGC-1*α* has anticancer effects. As described above, PGC-1*α* is decreased in colon [88], breast [89], and ovarian cancer cells [41], and PGC-1*α* overexpression in human ovarian cancer cell line Ho-8910 has been shown to induce apoptosis via downregulation of Bcl-1 and upregulation of Bax, suggesting that PGC-1*α* may be a contributor to the inhibition of tumor growth [41]. Lee et al. found that PPAR*γ* activation and PGC-1*α* overexpression by adenovirus infection in HepG2 human hepatoma cells induced E-cadherin upregulation and inhibited cell motility [42]. One report showed that PGC-1*α* overexpression induced apoptosis via ROS accumulation in HT29 and HCT116 colorectal cancer cells. In addition, PGC-1*α* overexpression reduced tumor growth in an HT29 xenograft model, suggesting a role of PGC-1*α* as a tumor suppressor [43]. Zhang et al. reported that von Hippel-Lindau- (VHL-) deficient clear cell renal carcinomas exhibited higher levels of HIF-1*α* and enhanced glycolysis [101]. HIF-1*α* is known to induce the expression of transcriptional repressor Dec1, which leads to the suppression of PGC-1*α* expression and the inhibition of mitochondrial respiration [102]. However, the enforced PGC-1*α* expression in VHL-deficient cells, despite the restoration of mitochondrial function, did not block the inhibition of cell growth and enhanced sensitivity to cytotoxic therapies in oxidative stress conditions [102]. This is in line with clinical clear cell carcinoma data that showed the correlation of higher mitochondrial mass with reduced tumor aggressiveness [103], and the association of lower PGC-1*α* levels with worse patient outcome [102]. It was shown that PGC-1*α* attenuates stress responses necessary for cancer cell survival, by interacting with heat-shock factor 1 [104]. Wang and Moraes revealed that increased PGC-1*α* expression due to treatment with PPAR panagonist (bezafibrate) increased mitochondrial biogenesis, resulting in an inhibition of cancer cell proliferation under glycolytic conditions and inhibition of invasion [44]. In addition, PGC-1*α* downregulation by miRNA-217 led to the promotion of cancer cell proliferation in breast cancer cells, suggesting a role of PGC-1*α* as a tumor suppressor [105]. Recently, Torrano et al. showed that PGC-1*α* suppresses metastasis of prostate carcinoma through an ERR*α*-dependent transcriptional program [45]. Highly metastatic melanoma cells expressed lower levels of PGC-1*α* [46, 106]. In turn, these PGC-1*α*-low cells expressed higher levels of integrin, TGF*β*, and Wnt signaling components involved in metastasis. It was shown that genetic depletion of PGC-1*α* increased metastasis in poorly invasive melanoma cells [46]. In contrast, PGC-1*α* overexpression in melanoma cells by ectopic expression or exposure to BRAF^V600E^ inhibitor vemurafenib suppressed metastasis through the direct regulation of inhibitor of DNA binding protein 2 (ID2) and inhibition of TCF-mediated gene transcription [46].

As described above, there have been many studies of the role of PGC-1*α* in tumor progression. However, it is still not sure if PGC-1*α* acts as a tumor promoter or tumor suppressor, and to date it is thought that its effect on tumor varies depending on the tissue context and tumor type (reviewed in [107]).

## 4. Conclusions

PPAR*γ* and PGC-1*α* are emerging proteins involved in tumorigenesis and attractive topics to study for further understanding of cancer biology. Originally, PGC-1*α* was identified as a PPAR*γ* interacting protein. However, most of the reported actions of PGC-1*α* in cancer were not related to the expression of PPAR*γ*. Despite the fact that PPAR*γ* and PGC-1*α* can each act as both tumor promoter and tumor suppressor, there is no clearly defined mechanism that can explain the contradictory dual effects. However, their dual actions can be explained, in part, by their cell type-specific effects and variable interacting proteins. Therefore, each of the molecular interactions of PPAR*γ* and PGC-1*α* with other transcriptional partners needs to be further investigated to understand the role of PPAR*γ* and PGC-1*α* in cancer.

## Figures and Tables

**Table 1:** The role and action mechanisms of PPAR*γ* as a tumor suppressor.

Experimental system		Role and action mechanisms	References
Modification	Cell type		
Troglitazone treatment (PPAR*γ* ligand)	Human colon cancer SW620, HCT116 cells	Inhibition of cell proliferation; induction of apoptosis; inactivation of NF-*κ*B by suppression of GSK-3*β*	[24]
PPAR*γ* activation by 4-O-methylhonokiol treatment	PC3, LNCap prostate cancer cells, PC3 xenografts	Inhibition of cell proliferation and tumor growth; induction of apoptosis; p21-mediated suppression of NF-*κ*B activity	[25]
PPAR*γ* overexpression	Human colon cancer HT-29 cells	Inhibition of cell proliferation and tumor growth; ubiquitination and degradation of NF-*κ*B by PPAR*γ*	[26]
PPAR*γ* overexpression	Human colon cancer HT-29 cells	Inhibition of cell proliferation; ubiquitination and degradation of MUC1-C by PPAR*γ*	[27]
PPAR*γ* activation by efatutazone treatment (third-generation PPAR*γ* agonist)	TE-4, TE-8, TE-11, TE-6 esophageal squamous cell carcinoma (ESCC) cells; TE-4 xenografts	Inhibition of cell proliferation and tumor growth; increased p21 protein levels by inactivation of Akt	[28]
Pioglitazone and 6-OH-11-O-hydroxy phenanthrene (PPAR*γ* and RXR agonist treatment)	Breast cancer MCF-7 cells, breast cancer associated fibroblast	Inhibition of cancer stem cell survival; inhibition of IL-6 promoter and reduced MMP-2, MMP-9 expression and activity	[29]
Pioglitazone treatment (PPAR*γ* ligand)	Chronic myeloid leukemia cells, leukemia stem cell (LSC)	Inhibition of cancer stem cell survival; decreased expression of STAT5 and HIF-1*α*	[30]
PPAR*γ* overexpression by PPAR*γ* plasmid	Gastric cancer cell lines (MKN-28, SGC-7901, BGC-823)	Inhibition of cell proliferation and migration; downregulation of TERT and ENAH by inhibition of *β*-catenin	[31]
PPAR*γ* activation by troglitazone, PPAR*γ* siRNA transfection	Human breast cancer cell lines (MCF-7, MDA-MB-231)	Inhibition of cell proliferation; upregulation of tumor suppressor *Cyld*	[32]
PPAR*γ* activation by rosiglitazone, PPAR*γ* inhibition by GW9662	Human breast cancer cell lines (MCF-7, MDA-MB-231)	Inhibition of cell migration and invasion; downregulation of CXCR4 gene expression	[33]

**Table 2:** The role and action mechanisms of PGC-1*α* in cancer.

Experimental system		Role and action mechanisms	References
Modification	Cell type		
*Tumor-promoting functions of PGC-1α*			
PGC-1*α* knockdown	Human prostate cancer PC3, LNCap cells	Stimulation of cell proliferation; activation of androgen receptor	[17]
Increased PGC-1*α* expression in arsenic-induced skin cancer	Skin cancer	Stimulation of cell proliferation; enhanced mitochondrial biogenesis	[34]
*Pgc-1α* knockout and knockdown by lentivirus-based PGC-1*α* shRNA	Human colorectal cancer cell line (Colo205)	Stimulation of carcinogenesis and tumor growth; induction of lipogenic enzymes	[35]
PGC-1*α* overexpression by PGC-1*α* plasmid	Human embryonic kidney cells, human colorectal cancer SNU-C4 cells, xenograft model	Stimulation of cell proliferation and tumorigenesis; upregulation of Sp1 and ACBP; upregulation of antioxidant enzyme (catalase, SOD)	[36]
PGC-1*α* shRNA knockdown	Human melanoma PGC-1*α*-positive A375 cells, xenograft model	Inhibition of apoptosis; decreased ROS production, induction of ROS detoxifying enzymes	[37]
Increased PGC-1*α* expression in breast cancer cell	Breast cancer cell	Stimulation of cell proliferation; enhanced glutamine-mediated lipid biosynthesis	[38]
*Pgc-1α* shRNA knockdown	Human prostate cancer cell line (C4-2 cells)	Stimulation of cell proliferation; increased mitochondrial biogenesis	[39]
PGC-1*α* shRNA knockdown and PGC-1*α* overexpression	Human breast cancer cell, human melanoma cells	Stimulation of cell proliferation, increased invasion; increased mitochondrial biogenesis and oxidative phosphorylation	[40]
*Anticancer functions of PGC-1α*			
PGC-1*α* overexpression by adenovirus infection	Human ovarian cancer cell line (Ho-8910)	Induction of apoptosis; downregulation of Bcl-2 and upregulation of Bax	[41]
PGC-1*α* overexpression by adenovirus infection	Human hepatoma cell line (HepG2)	Inhibition of cell motility; upregulation of E-cadherin	[42]
PGC-1*α* overexpression	Human colorectal cancer cell lines (HT29 and HCT116)	Induction of apoptosis; ROS accumulation	[43]
Increased expression of PGC-1*α* by bezafibrate (PPAR panagonist)	Human cancer cell lines (HeLa, 143B, MDA-MB-231)	Inhibition of cell proliferation and invasion; increased mitochondrial biogenesis	[44]
PGC-1*α* overexpression	Human prostate cancer cell	Inhibition of cell proliferation and inhibition of metastasis; activation of ERR*α*-dependent transcriptional program; induction of catabolic state	[45]
PGC-1*α* overexpression by adenovirus infection, CRISPR-mediated PGC-1*α* depletion	Human melanoma cell	Inhibition of metastasis; inhibition of inhibitor of DNA binding protein 2 (ID2) and TCF-mediated gene transcription	[46]
